# Serum alanine aminotransferase activity and risk factors for cardiovascular disease in a Caucasian population: the Tromsø study

**DOI:** 10.1186/s12872-020-01826-1

**Published:** 2021-01-13

**Authors:** Svein Ivar Bekkelund

**Affiliations:** 1 Department of Clinical Medicine, UiT – The Arctic University of Norway, 9037 Tromsø, Norway; 2 Department of Neurology, University Hospital of North Norway, 9038 Tromsø, Norway

**Keywords:** Alanine aminotransferase, Sex difference, Age, BMI, Population study

## Abstract

**Background:**

High and low levels of serum alanine aminotransferase (ALT) are both associated with cardiovascular diseases (CVD) risks especially in elderly, but the mechanisms are less known. This study investigated associations between ALT and CVD risk factors including effects of sex and age in a Caucasian population.

**Methods:**

Cross-sectional data were analysed sex-stratified in 2555 men (mean age 60.4 years) and 2858 women (mean age 60.0 years) from the population study Tromsø 6. Associations were assessed by variance analysis and multivariable logistic regression of odds to have abnormal ALT. Risk factors included body mass index (BMI), waist-to-hip-ratio, blood pressure, lipids, glucose, glycated haemoglobin and high-sensitive C-reactive protein (CRP).

**Results:**

Abnormal elevated ALT was detected in 113 men (4.4%) and 188 women (6.6%). Most CVD risk factors associated positively with ALT in both sexes except systolic blood pressure and CRP (women only), while ALT was positively associated with age in men when adjusted for CVD risk factors, *P* < 0.001. BMI predicted ALT in men (OR 0.94; 95% CI 0.88–1.00, *P* = 0.047) and women (OR 0.90; 95% CI 0.86–0.95, *P* < 0.001). A linear inversed association between age and ALT in men and a non-linear inversed U-trend in women with maximum level between 60 and 64 years were found.

**Conclusion:**

This study confirms a positive relationship between ALT and CVD risk factors, particularly BMI. Age is not a major confounder in the ALT-CVD relationship, but separate sex-analyses is recommended in such studies.

## Background

Elevated levels of alanine aminotransferase (ALT) is associated with higher risk to develop cardiovascular disease (CVD) [1, 2], obesity [3, 4], insulin resistance [5], the metabolic syndrome and type 2 diabetes [6, 7]. The full spectre of underlying mechanisms is unresolved, but ALT-related non-alcoholic fatty liver disease (NAFLD) is an important condition involved, at least in men [8, 9]. Higher prevalence of metabolic abnormalities in men may in part explain sex-differences in associations between ALT and metabolic syndrome [10]. Low levels have also been reported to be unfavourable and may be explained by higher age, sarcopenia and hepatic ageing [11]. Accordingly, a J-shaped ALT-mortality curve is demonstrated [12–14]. An American survey with measurement of body composition in 15,028 inhabitants reported higher mortality risk in subgroups with low ALT (deciles 1–3) and an insignificantly increased mortality rate in those with the highest ALT level (decile 10) [14]. The risk excess associated with lower ALT levels was probably due to decreased appendicular lean mass found in the same study fraction [14]. Furthermore, ALT-related coronary disease may be unrelated to presence of risk factors [15, 16]. Whether this may be explained in part by age and sex variations is unknown.

An inversed relationship between age and ALT previously reported [17] has been associated with male sex in particular [4]. Israeli and Italian cohorts of men and women recruited from general practice populations demonstrated an inversed U-shaped relationship between age and ALT [18, 19]. The purposes of the present study were to investigate associations between ALT and CVD risk factors, identify factor(s) most strongly associated with ALT in a Scandinavian predominantly Caucasian general population and clarify the effects of sex and age.

## Methods

### Patients and ethics

The participants of this cross-sectional community study were recruited from the 6^th^ Tromsø Study [20]. ALT was measured in 5413 participants (2555 men and 2858 women), aged 30–87 years, representing 42% of those attended. The ethnic origin included 87.3% Norwegians, 1.6% Sami and 1.3% of Finnish origin, 2.2% of other ethnicities, and 7.6% with unknown ethnicity. The data were collected from October 2007 to September 2008.

### Measurements

Clinical examinations, body mass index (BMI) and blood pressure are performed standardized in the Tromsø study [21]. Self-reported reported previous heart disease was registered via standard questionnaires in the Tromsø study and diabetes defined as glycated haemoglobin (HbA_1c_) ≥ 6.5%.

ALT, aspartate aminotransferase (AST) and gamma glutamyl transferase (GGT), HbA1c, serum-glucose, total cholesterol, high-density lipoprotein (HDL), low density lipoprotein (LDL) and high-sensitive C-reactive protein (hs-CRP) were analyzed using standard methods at Department of Clinical Biochemistry, University Hospital of North Norway, Tromsø [22]. The standard cut-off limits for ALT, used in the Hospital are those developed by the Nordic Reference Interval Project (NORIP) [23]. Serum ALT reference limits were 10–70 U/L for men and 10–45 U/L in women. Total cholesterol/HDL cholesterol ratio was used in the statistical analyses.

### Statistical analysis

ALT values showed left-sided skewness by histograms and therefore log-transformed in the analyses. Additionally, AST, GGT, hs-CRP, HbA_1c_ and glucose-values were log-transformed due to right-sided skewness. Descriptive data are presented as mean and standard deviations (SD) for continuous variables or numbers and frequencies for dichotomous data. Spearman´s correlation coefficient was calculated for associations between continuous variables. Two-sided student´s t-test was used to compare means respectively χ^2^-test between frequencies of data and ANOVA used to test differences between means of ALT vs. age quartiles and independent variables vs. ALT quartiles while associations between age and ALT adjusted for covariates was done by ANCOVA. By multiple logistic regression analysis, possible confounders were tested and adjusted for with abnormal ALT as dependent variable and age, BMI, waist-to-hip-ratio, diastolic blood pressure, total/HDL cholesterol ratio, triglycerides and log CRP as independent variables; i.e. those variables found statistically significantly associated with ALT in the variance analyses. Systolic blood pressure is not included in the female model due to high correlation with diastolic blood pressure (r = 0.651, *P* < 0.001). *P* < 0.05 was considered statistically significant and SPSS software (Statistical Package for Social Science INC, Chicago, Illinois, USA), version 26) was used in the statistical analyses.

## Results

### Alanine aminotransferase, supplementary blood samples and clinical characteristics in men and women

Table 1 shows characteristics of the subjects in the population. Men had higher levels of liver markers and CVD risk markers including BMI and waist-to-hip-ratio but lower total cholesterol, LDL and HDL levels than women (Table 1). Accordingly, 580 (20.3%) women used lipid lowering drugs compared to 185 (7.2%) in the mail group, *P* < 0.001. The rate of hypertension was high in the total group, but relatively higher in men (Table 1).

**Table 1:** Basic characteristics and endpoints of the participants presented as mean (SD) or numbers (%)

Variables	Men (*n* = 2555)	Women (*n* = 2858)	*P* value
Age (year)	60.4 (12.2)	60.0 (12.8)	0.27
BMI (kg/m^2^)	27.3 (3.7)	26.7 (4.6)	< 0.001
Waist-to-hip-ratio	0.95 (0.06)	0.87 (0.07)	< 0.001
Obesity (BMI ≥ 30 kg/m^2^)	526 (20.6)	595 (20.8)	0.81
Heavy exerciser^†^	431 (16.9)	433 (15.2)	0.056
Alcohol 2–3 times or more per week	595 (23.3)	511 (17.9)	< 0.001
Heavy alcohol drinker^‡^	72 (2.8)	15 (0.5)	< 0.001
Systolic blood pressure (mmHg)	140.6 (21.0)	138.2 (25.7)	< 0.001
Diastolic blood pressure (mmHg)	81.7 (10.3)	76.0 (10.3)	< 0.001
Hypertension	1275 (49.9)	1293 (45.2)	0.001
Diabetes	151 (5.9)	130 (4.5)	0.031
Coronary heart disease	252 (9.9)	86 (3.0)	< 0.001
Total cholesterol (mmol/L)	5.50 (1.08)	5.81 (1.11)	< 0.001
HDL cholesterol (mmol/L)	1.37 (0.38)	1.66 (0.44)	< 0.001
LDL cholesterol (mmol/L)	3.54 (0.95)	3.63 (0.98)	0.001
Total/HDL cholesterol ratio	4.29 (1.36)	3.71 (1.13)	< 0.001
Triglycerides (mmol/L)	1.66 (0.99)	1.43 (0.80)	< 0.001
S-ALT (U/L)	32.62 (19.4)	26.30 (25.61)	< 0.001^§^
High s-ALT (U/L)	113 (4.4)	188 (6.6)	0.001
S-AST (U/L)	27.85 (9.96)	25.25 (14.15)	< 0.001^§^
S-GGT (U/L)	37.17 (39.16)	27.39 (37.95)	< 0.001^§^
S-creatinine (µmol/L)	77.91 (16.01)	61.83 (12.83)	< 0.001
Hs-CRP (mg/dl)	2.66 (5.17)	2.76 (5.76)	0.59
S-glucose (mmol/L)	5.42 (1.31)	5.17 (1.11)	< 0.001^§^
HbA_1C_ (%)	5.78 (0.73)	5.69 (0.63)	< 0.001^§^

*ALT* alanine aminotransferase, *AST* aspartate aminotransferase, *BMI* body mass index, *HbA*_*1C*_ glycated haemoglobin, *GGT* gamma glutamyl transferase, *HDL* high density lipoprotein, *Hs-CRP* high-sensitive C-reactive protein, *LDL* low density lipoprotein, *SD* standard deviation

^†^Moderate or hard leisure exercise ≥ 2 h per week

^‡^Drinking ≥ 6 alcohol units ≥ 2 times per week

^§^Analyzed log-transformed

### Alanine aminotransferase is associated with cardiovascular risk factors

Tables 2 and 3 show positive and significant relationships between ALT and BMI, waist-to-hip-ratio and most CVD-related variables when comparing lowest and highest quartiles of variables. Exceptions are insignificant associations with diastolic blood pressure and CRP in men (Table 2). Thus, CRP-levels were elevated in 1st ALT-quartiles in both sexes and thereby slightly J-shaped. Consequently, the difference between means of ALT in 2 and 4. Quartiles in men was significant (*P* = 0.03).

**Table 2:** Age and cardiovascular risk factors in relation to quartiles of log ALT in men (mean and SD or numbers and %)

Variables	Log ALT quartiles				P (trend)
Log ALT (U/L)	< 1.32	1.32 – 1.44	1.45 – 1.58	≥ 1.58	
N	631	662	622	637	
Age (years)	64.1 (12.4)	61.1 (12.3)	59.6 (11.5)	56.5 (11.6)	< 0.001
BMI (kg/m^2^)	25.3 (3.3)	26.6 (3.2)	27.9 (3.5)	29.1 (5.0)	< 0.001
Waist-to-hip-ratio	0.93 (0.07)	0.95 (0.06)	0.95 (0.06)	0.98 (0.06)	< 0.001
Systolic blood pressure (mmHg)	140.0 (22.0)	140.1 (20.7)	141.7 (20.8)	140.8 (20.5)	0.50
Diastolic blood pressure (mmHg)	79.2 (10.0)	81.1 (10.1)	82.4 (10.0)	84.0 (10.6)	< 0.001
Total/HDL cholesterol ratio	3.88 (1.24)	4.15 (1.31)	4.35 (1.32)	4.73 (1.43)	< 0.001
Triglycerides (mmol/L)	1.39 (0.75)	1.55 (0.99)	1.72 (0.96)	1.97 (1.16)	< 0.001
Log hs-CRP (mg/dl)	0.16 (0.48)	0.13 (0.42)	0.15 (0.39)	0.22 (0.40)	0.10
Log s-glucose (mmol/L)	0.71 (0.07)	0.72 (0.07)	0.73 (0.07)	0.74 (0.09)	< 0.001
Log HbA_1C_ (%)	0.76 (0.05)	0.76 (0.04)	0.76 (0.04)	0.77 (0.05)	0.007
Heavy exerciser†	98 (15.5)	131 (19.8)	104 (16.7)	98 (15.4)	0.64
Alcohol 2–3 times per week	134 (21.2)	159 (24.0)	146 (23.5)	156 (24.5)	0.24

*ALT* alanine aminotransferase, *BMI* body mass index, *HbA*_*1C*_ glycated haemoglobin, *HDL* high density lipoprotein, *Hs-CRP* high-sensitive C-reactive protein, *SD* standard deviation

^†^Moderate or hard leisure exercise ≥ 2 h per week

**Table 3:** Age and cardiovascular risk factors in relation to quartiles of log ALT in women (mean and SD or numbers and %)

Variables	Log ALT quartiles				P (trend)
Log ALT (U/L)	< 1.23	1.23 – 1.34	1.35–1.46	> 1.46	
N	717	745	684	712	
Age (years)	56.3 (15.0)	60.8 (12.7)	61.9 (11.6)	60.9 (10.8)	< 0.001
BMI (kg/m^2^)	25.2 (3.8)	26.1 (4.3)	27.0 (4.6)	28.4 (4.9)	< 0.001
Waist-to-hip-ratio	0.86 (0.07)	0.86 (0.07)	0.87 (0.06)	0.89 (0.07)	< 0.001
Systolic blood pressure (mmHg)	132.1 (26.0)	138.3 (25.8)	139.7 (25.0)	144.9 (25.9)	< 0.001
Diastolic blood pressure (mmHg)	74.3 (10.6)	75.5 (10.2)	76.1 (10.0)	78.8 (11.1)	< 0.001
Total/HDL cholesterol ratio	3.51 (1.06)	3.59 (1.00)	3.70 (1.16)	4.17 (1.28)	< 0.001
Triglycerides (mmol/L)	1.27 (0.65)	1.31 (0.66)	1.43 (0.78)	1.70 (1.00)	< 0.001
Log hs-CRP (mg/dl)	0.13 (0.49)	0.10 (0.44)	0.14 (0.43)	0.24 (0.41)	< 0.001
Log s-glucose (mmol/L)	0.69 (0.07)	0.70 (0.06)	0.71 (0.06)	0.72 (0.08)	< 0.001
Log HbA_1C_ (%)	0.74 (0.04)	0.75 (0.04)	0.75 (0.04)	0.76 (0.05)	< 0.001
Heavy exerciser†	94 (13.1)	126 (16.9)	113 (16.5)	43 (6.0)	0.85
Alcohol ≥ 2–3 times per week	122 (17.0)	139 (18.7)	120 (17.5)	127 (17.8)	0.88

*ALT* alanine aminotransferase, *BMI* body mass index, *HbA*_*1C*_ glycated haemoglobin, *HDL* high density lipoprotein, *Hs-CRP* high-sensitive C-reactive protein, *SD* standard deviation

^†^Moderate or hard leisure exercise ≥ 2 h per week

### Age- and sex are differently associated with serum alanine aminotransferase levels

Spearman´s correlation coefficient was inversed in the group of 2555 men (r = − 0.231, *P* < 0.001) and positive in 2858 women (r = 0.124, *P* < 0.001). A negative linear association between age quintiles and log ALT was found for men and a non-linear inversed U-shaped relationship with maximum level between 60 and 64 years was observed in women in a variance analysis adjusted for covariates. Thus, mean adjusted log ALT in men changed from 1.51 U/L from the youngest group to 1.37 U/L in the oldest (9.3% decline) (*P* < 0.001). Adjusted log ALT in women: 1.30 (< 45 years), 1.38 (45–59 years), 1.40 (60–64 years), 1.38 (65–70 years) and 1.33 (≥ 70 years).

### BMI independently predicted ALT in men and women

Variables included in the multivariate analysis are those significantly associated with log ALT by ANOVA (Tables 2 and 3) except systolic blood pressure in women. BMI was the only predictor independently associated with log ALT in both sexes after adjusting for age, lipids, glucose, HbA_1C_ (%) (men and women) and log CRP in women (Table 4).

**Table 4:** Multivariable logistic regression of independent variables with odds (OR) of having ALT above upper reference limit in men (> 70 U/L) and women (> 45 U/L)

Predictors	Abnormal ALT, men (n = 113)		Abnormal ALT, women (n = 188)	
	OR (95% CI)	*P* value	OR (95% CI)	*P* value
Age (years)	1.06 (1.04, 1.08)	< 0.001	1.01 (0.99, 1.03)	0.43
BMI (kg/m^2^)	0.94 (0.88, 1.00)	0.047	0.90 (0.86, 0.95)	< 0.001
Waist-to-hip-ratio	0.003 (0.00, 0.09)	0.001	0.11 (0.002, 10.55)	0.37
Diastolic blood pressure (mmHg)	0.98 (0.96, 1.00)	0.068	1.00 (0.95, 1.00)	0.025
Total/HDL cholesterol ratio	1.01 (0.84, 1.22)	0.93	0.81 (0.62, 1.06)	0.12
Triglycerides (mmol/L)	0.93 (0.74, 1.17)	0.54	1.13 (0.79, 1.62)	0.50
Log s-glucose (mmol/L)	0.14 (0.01, 2.99)	0.21	0.21 (0.04, 10.68)	0.43
Log HbA_1C_ (%)	0.03 (0.00, 4.57)	0.17	0.01 (0.00, 7.77)	0.18
Log hs-CRP (mg/dl)			0.75 (0.40, 1.42)	0.38

*ALT* alanine aminotransferase, *BMI* body mass index, *HbA*_*1C*_ glycated haemoglobin, *Hs-CRP* high-sensitive C-reactive protein, *SD* standard deviation

## Discussion

ALT is associated with metabolic components and clinical CVD risk factors, but BMI was the only one independently associated with ALT in both sexes in a multivariate analysis. The relationship between age and ALT showed an inverted U-wave along quantiles of age-groups in women but was linearly and negative in men. Age did only represent a minor impact on the ALT-CVD connection, but only in men.

The positive relationship between ALT and CVD risk factors was independent of sex but age was a weak predictor of elevated ALT in men. The role of ALT in the pathophysiology of CVD is complex [24, 25]. Likewise, how age and sex act as possible confounders are undetermined. Thus, a recent study by Visaria et al. demonstrated increased overall mortality, including CVD-related mortality, to be associated with lower ALT quartiles in both sexes. However, when adjusted for covariates, an independent relation between ALT and CVD-related mortality remained evident in women but not in men [26]. A linear and inversed relationship between age and ALT in both sexes is reported in cross-sectional and longitudinal studies [17, 27]. An Australian community study in 1673 elderly men (≥ 70 years) designed as a survival analyses, showed an inversed relationship between age and ALT like the findings in the male group here [28]. That study found low ALT to be associated with reduced survival partly due to high age, but the mechanisms is otherwise largely unknown [28]. However, obesity without accompanied CVD has been related to increased survival (the obesity paradox) [29]. Like the present study, age and ALT was negatively correlated in men contrasting the positive relationship in women. Accordingly, the third U.S. National Health and Nutrition Examination Survey (n = 5724) reported positive associations between age and male sex in relation to ALT which abolished in a multivariate analysis. Also, BMI remained significantly associated with ALT [4]. Age, sex and BMI also influenced the transaminase levels in children and adolescents. In girls, ALT declined from infancy until 4 years of age, then increased to peak at 16 years. The same U-form was found for boys although a little delayed [30]. Boys had higher ALT levels than girls, and BMI correlated stronger with ALT in boys [30].

Despite different age-ALT curves between men and women in the present study, the relationships between ALT and CVD risk factors including glucose were approximately the same. In women, an inversed U-form with significant and opposite directed age-ALT correlations on both sides of the 60-year cut-off is demonstrated. A similar pattern with ALT-maximum at 65-year of age was found equal for both sexes in a recent cross-sectional study in 10,000 non-diabetic subjects [11]. By showing a similar trend for age vs. fasting glucose levels, glucose was suggested to entail a joint effect on the ALT levels across ages [11]. The same phenomenon is demonstrated in a study that included subjects living in aged home and participants recruited from three general practices (*n* = 335). ALT values was distributed along an inversed U-shaped curve with a peak level between 40 and 55 years [19]. In parallel, ALT increased until the third decade in men and the fifth decade in women in a large Italian study where ALT additionally associated positively with BMI, glucose and lipids [18].

The pattern of sex-difference in ALT vs. age in the present study is not shown in previous studies, but the data otherwise do not indicate any certain mechanism. A study in an obese cohort from the same area, showed an association between ALT and the muscular component of body composition in both sexes, but ALT was associated with fat mass in men only [22]. Furthermore, ALT was positively correlated with glucose, glycated haemoglobin and cholesterol in obese women [22]. ALT is higher in men than women but whether this is due to different body composition, sex hormones or confounders is unknown [31, 32]. Thus, ALT predicted prevalent coronary heart disease in men according to an European-American study [33].

### Limitations to the study

Although the Tromsø study provides robust data, the evidence of the present cross-sectional study may be questioned by low numbers of cases with abnormal elevated ALT used in the logistic regression analyses. Consequently, the power might have been insufficient in order to detect correct connections between biological variables in that part of the analyses (statistical type 2 error). On the other hand, the low correlation coefficients between ALT and age along with highly significant P-values in both men and women may reflect a large sample size with risk of false positive results. Further, the cross-sectional design makes conclusions about causality impossible. Nevertheless, the data may provide evidence to make hypothesis about sex-differences in ALT functions more specific.

## Conclusions

ALT is positively associated with CVD risk factors in both sexes. The data especially confirms an independent and equal association between ALT and BMI. The sex-specific variations in ALT levels seem to be unrelated to CVD-related risk factors and is largely unexplained. How age and sex influence upon the ALT-CVD relationship should be further investigated.

## Data Availability

Due to ethical and legal restrictions, the data set is only available upon request to the Tromsø Study. Authors given permission to analyze data are not allowed to make data sets publicly available. Any enquiries should be sent to the Institutional Data Access committee of The Tromsø study, Department of Community Medicine, Faculty of Health Sciences, UiT, The Arctic University of Tromsø (tromsous@uit.no).

## Abbreviations

ALT: Alanine aminotransferase
ANCOVA: Analysis of covariance
ANOVA: Analysis of variance
AST: Aspartate aminotransferase
BMI: Body mass index
CVD: Cardiovascular disease
CI: Confidence interval
HbA_1C_: Glycated haemoglobin
GGT: Gamma glutamyl transferase
HDL: High density lipoprotein
Hs-CRP: High-sensitive C-reactive protein
LDL: Low density lipoprotein
NAFLD: Non-alcoholic fatty liver disease
NORIP: Nordic Reference Interval Project
OR: Observed risk
REC: Regional ethical committee
SD: Standard deviation
